# Lamin B1 Accumulation’s Effects on Autosomal Dominant Leukodystrophy (ADLD): Induction of Reactivity in the Astrocytes

**DOI:** 10.3390/cells10102566

**Published:** 2021-09-28

**Authors:** Stefano Ratti, Isabella Rusciano, Sara Mongiorgi, Irene Neri, Alessandra Cappellini, Pietro Cortelli, Pann-Ghill Suh, James A. McCubrey, Lucia Manzoli, Lucio Cocco, Giulia Ramazzotti

**Affiliations:** 1 Cellular Signalling Laboratory, Department of Biomedical and NeuroMotor Sciences (DIBINEM), University of Bologna, 40126 Bologna, Italy; stefano.ratti@unibo.it (S.R.); isabella.rusciano3@unibo.it (I.R.); s.mongiorgi@unibo.it (S.M.); irene.neri2@studio.unibo.it (I.N.); alessandra.cappellini@unibo.it (A.C.); giulia.ramazzotti@unibo.it (G.R.); 2 Department of Biomedical and NeuroMotor Sciences (DIBINEM), University of Bologna, 40139 Bologna, Italy; pietro.cortelli@unibo.it; 3 IRCCS Istituto delle Scienze Neurologiche di Bologna, UOC NeuroMet, 40139 Bologna, Italy; 4 Korea Brain Research Institute, Daegu 41062, Korea; pgsuh@kbri.re.kr; 5 School of Life Sciences, Ulsan National Institute of Science and Technology (UNIST), Ulsan 689-798, Korea; 6 Department of Microbiology and Immunology, Brody School of Medicine at East Carolina University, Greenville, NC 27834, USA; mccubreyj@ecu.edu

**Keywords:** Lamin B1, ADLD, reactive astrocyte, cell survival, apoptosis, cell cycle, cell proliferation, cell viability, cytotoxicity

## Abstract

Autosomal dominant leukodystrophy (ADLD) is an extremely rare and fatal neurodegenerative disease due to the overexpression of the nuclear lamina component Lamin B1. Many aspects of the pathology still remain unrevealed. This work highlights the effect of Lamin B1 accumulation on different cellular functions in an ADLD astrocytic in vitro model. Lamin B1 overexpression induces alterations in cell survival signaling pathways with GSK3β inactivation, but not the upregulation of β-catenin targets, therefore resulting in a reduction in astrocyte survival. Moreover, Lamin B1 build up affects proliferation and cell cycle progression with an increase of PPARγ and p27 and a decrease of Cyclin D1. These events are also associated to a reduction in cell viability and an induction of apoptosis. Interestingly, ADLD astrocytes trigger a tentative activation of survival pathways that are ineffective. Finally, astrocytes overexpressing Lamin B1 show increased immunoreactivity for both GFAP and vimentin together with NF-kB phosphorylation and c-Fos increase, suggesting astrocytes reactivity and substantial cellular activation. These data demonstrate that Lamin B1 accumulation is correlated to biochemical, metabolic, and morphologic remodeling, probably related to the induction of a reactive astrocytes phenotype that could be strictly associated to ADLD pathological mechanisms.

## 1. Introduction

**Figure 1:**
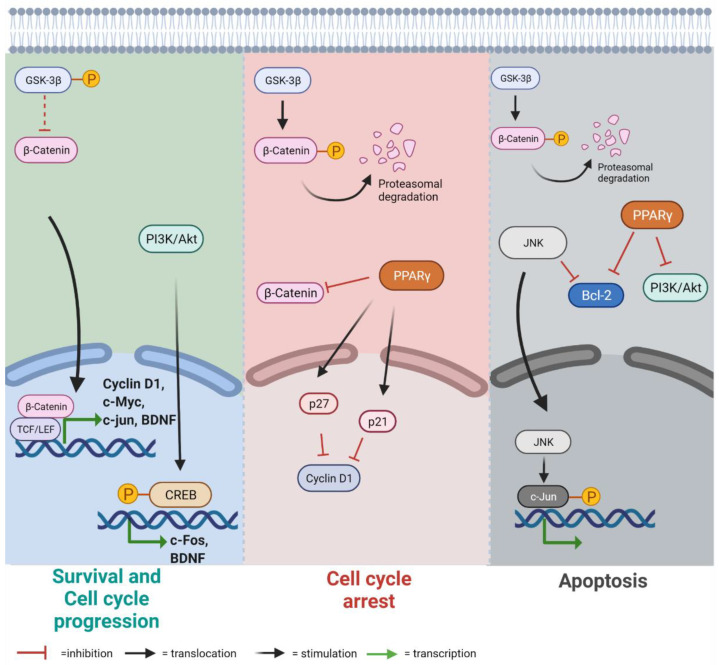
Schematic representation of the signaling pathways involved in survival and cell cycle progression, cell cycle arrest, and apoptosis affected by Lamin B1 overexpression. Created with Biorender.com.

Adult onset autosomal dominant leukodystrophy (ADLD) is an extremely rare, fatal and late onset neurological disorder, characterized by a progressive loss of white matter in the central nervous system (CNS) [1,2,3]. The disease manifests itself in the fourth to fifth decades of adulthood without any described gender difference. The real prevalence of this fatal pathology still remains uncertain, with sporadic new clinical case reports from different geographical areas suggesting a possible heterogeneity in the first clinical manifestations and signs [4,5,6,7,8,9]. In majority of ADLD cases, the first clinical manifestations are related to autonomic dysfunction, followed by ataxia and cognitive impairment that signal pyramidal and cerebellar involvement. Genetically, ADLD is characterized by alterations of the LMNB1 gene (chr5q23.2), resulting in the accumulation of Lamin B1, a component of the nuclear lamina [1,8,10]. Demyelination seems to be one of the most significant aspects of ADLD, even if the molecular aspects detailing how the genetic alterations affect the cellular mechanisms to drive the onset and development of this pathology are still not entirely known. Moreover, the mechanisms regulating the myelination are still not completely defined. Oligodendrocyte cells are certainly the primary cells responsible for the myelination of the axons [11], but signals deriving from astrocytes and neurons are also essential to promote myelin sheath formation and its maintenance during life [12]. Astrocytes, in particular, play an important role in supporting myelination [13,14]. Indeed, it has been shown that astrocytes overexpressing LMNB1 and cells from ADLD patients show several nuclear alterations not present in oligodendrocytes overexpressing LMNB1 [15]. Furthermore, the accumulation of Lamin B1 in astrocytes induces a reduction in Leukemia Inhibitory Factor-like protein (LIF) secretion, leading to the downregulation of Jak/Stat3 and PI3K/Akt axes. These pathways are essential for astrocytes survival, and their inactivation would lead to a reduction of the astrocytes’ fundamental support to oligodendrocytes in the myelination process. The development of many neurological disorders such as Parkinson’s, Alzheimer’s, schizophrenia, amyotrophic lateral sclerosis, bipolar disorder, and mood disorders has been associated to the aberrant activity of Glycogen Synthase Kinase-3 (GSK-3) [16]. GSK-3 is a protein serine/threonine kinase that has a central role in several cellular signaling pathways through the modulation of many transcription factors, such as c-Myc in proliferation, Bcl2, and CREB in survival, cell cycle, and apoptosis regulation [17,18]. When GSK-3 is phosphorylated, and therefore inactivated, β-catenin can translocate to the nucleus and bind the T-cell factor/lymphoid enhancer factor (TCF/LEF) [19], inducing cyclin D1 and transcription factors such as c-Myc and c-Jun, all involved in cell division, proliferation, invasion, and stem cell maintenance [20]. In several diseases, such as gliomas, neurodegenerative diseases, amyotrophic lateral sclerosis, and multiple sclerosis, the Wnt/β-catenin pathway acts in an opposite way of peroxisome proliferator-activated receptor gamma (PPARγ) [21]. Several studies have demonstrated that PPARγ overexpression stimulates the expression of cyclin-dependent kinase inhibitors p27 and p21, stopping the cytosolic β-catenin accumulation and decreasing the expression of cyclin D1, promoting cell cycle arrest [20]. Moreover, PPARγ agonists can downregulate Bcl2 [22] and PI3K/Akt pathway, promoting apoptosis [23]. Among apoptotic signaling pathways, Jun N-terminal kinases (JNKs) play an important role in the regulation of pro-apoptotic proteins implicated in the activation of the extrinsic and intrinsic apoptotic pathways [24]. It has been observed that JNK is required for the apoptosis of neurons in the central nervous system, and that the expression of dominant negative inhibitors of nuclear JNK confers resistance to apoptosis following the withdrawal of trophic support [25]. Proliferation, differentiation, survival, and neurogenesis have also been correlated with the activity of CREB [26]. Several studies have demonstrated that knocking down CREB expression in the neurons of developing CNS, significantly decreases the transcription of many CRE-regulated genes, such as Bcl2 [27], leading to apoptosis and the postnatal ablation of genes, which leads to neuronal degeneration [28]. CREB represents a downstream target of the neurotrophin brain-derived neurotrophic factor (BDNF) [26], which promotes the development of specific neuronal cells and confers neuroprotection under several conditions [29]. Dysfunction of BDNF signaling causes deficits in neuronal growth and synaptic transmission, leading to disorganized brain functions. Loss of CREB/BDNF signaling impairs neurodevelopment, but CREB upregulation causes aberrant signal transduction that represents the hallmarks of some brain tumors, determining the altered regulation of proliferation, apoptosis, metastasis, and metabolism [26]. The schematic in Figure 1 shows and summarizes the main signaling pathways mentioned above.

Only recently have astrocytes been widely considered to give an important contribution to neurological conditions, as opposed to the classical representation of these cells as bystanders in neuroinflammatory diseases. Astrocytes that change their morphology, and/or protein expression and secretion, and metabolic activity in response to injury of the CNS due to different noxae are called “reactive astrocytes” [30]. Nonetheless, defined guidelines to identify categories of reactive astrocytes and to classify them are still lacking. For the time being, the upregulation of intermediate filaments proteins, such as GFAP, vimentin, synemin, and nestin, the increase in several enzymes involved in metabolic processes, and the increase in protein secretion and nuclear translocation of transcription factors, such as NF-kB, are all considered potential markers of reactive astrocytes [30,31].

In this work, we analyzed, for the first time in an experimental in vitro astrocytic model of ADLD, the effect of Lamin B1 accumulation on the induction of astrocytes’ reactivity, focusing on pivotal cellular mechanisms that might be involved in the development of this very rare and intriguing pathology. 

## 2. Materials and Methods

### 2.1. Cell Culture and Lentiviral Transduction 

U87-MG (HTB-14 ATCC, Old Town Manassas, VA, USA) glioblastoma-astrocytoma cell line was cultured in Eagle’s Minimum Essential Medium (EMEM) (Corning, New York, NY, USA) supplemented with 10% heat-inactivated fetal bovine serum (FBS) and 1% Penicillin/Streptomycin (both from Sigma-Aldrich, St. Louis, MO, USA). 

HEK 293T human embryonic kidney cells (Genecopoeia Inc., Rockville, MD, USA) were cultured in DMEM (Corning) supplemented with 10% FBS and 1% penicillin/streptomycin, while 5% FBS was used during transfection. 

All cells were maintained in a humidified 5% CO_2_ incubator at 37 °C.

EX-I3724-Lv201 coding for Homo Sapiens LMNB1 and EX-NEG-Lv201 empty control vectors (Genecopoeia), respectively, were used to produce lentiviruses used to overexpress *LMNB1* and green fluorescent protein (GFP), as well as lentiviruses coding only for GFP, as control. HEK293T cells were used as the viral packaging system using the Lenti-Pac HIV expression packaging kit (Genecopoeia) according to manufacturer’s protocol. 

To perform viral transduction, U87-MG cells were plated the day before infection at 5 × 10^5^ cells/well of a 6-well plate. The next day, virus supernatants were added with polybrene 8µg/mL and applied to cultured cells. The supernatants were replaced with fresh media the next day and 48 h after transduction 2 µg/mL of puromycin (Sigma-Aldrich) was added to the medium.

### 2.2. RNA Extraction, Reverse Transcription and Real-Time PCR

RNeasy Mini Kit (Qiagen, Hilden, Germany) was used to extract total RNA from samples and extracted RNA was quantified at the Nanodrop spectrophotometer. Precisely 1 µg of total RNA was reverse transcribed into cDNA using TaqMan Reverse Transcription Reagents (Thermo Fisher Scientific, Waltham, MA, USA), following the manufacturer’s protocol.

mRNA expression levels were detected by performing qPCR on 100 ng of cDNA per reaction in the QuantStudio 1 Real-Time PCR System (Thermo Fisher Scientific), using TaqMan Universal Master mix II (Thermo Fisher Scientific) and TaqMan probes. The following validated gene expression assays were used: LMNB1 Hs.PT.58.40133522 and GAPDH Hs.PT.39a.22214836 (IDT, Coralville, IA, USA); TCF7 Hs00175273_m1 and BNDF Hs02718934_s1 (Thermo Fisher Scientific). 

### 2.3. Protein Extraction, Nuclear Protein Extraction and Western Blot

Whole cell lysates which were obtained by lysing cells were supplemented in T-PER lysis buffer with Halt protease and phosphatase inhibitor cocktails (all from Thermo Fisher Scientific) and sonicated for 15 s at a power of 40–50%. Nuclear protein fraction was isolated by using NE-PER nuclear and cytoplasmic extraction kit (Thermo Fisher) following the manufacturer’s instruction.

Cell lysates were quantified with the Bradford Protein Assay (Bio-Rad Laboratories, Hercules, CA, USA). Equal amounts of total protein lysates were separated on Bolt 4–12%, polyacrylamide-0.1% SDS gels (Thermo Fisher Scientific) and transferred onto nitrocellulose membrane. Membranes were washed with PBS-0.1% Tween-20 (PBST) and blocked with 5% w/v non-fat dry milk in PBST for 1 h at room temperature. Next, membranes were incubated with primary antibodies overnight at 4 °C. Then, blots were incubated with the corresponding peroxidase-conjugated secondary antibodies (Thermo Fisher Scientific) diluted in PBST for 1 h at room temperature. ECL enhanced chemiluminescence reagents (Thermo Fisher Scientific) were used to detect immunoreactive bands and images captured with the iBright Imaging system (Thermo Fisher Scientific). Samples were normalized by using the iBright software that allows to quantitate the total protein signal in each lane after membrane staining with the No-Stain Protein Labeling Reagent (Thermo Fisher Scientific). 

### 2.4. Immunocytochemistry

Cells were grown on coverslips and fixed in ice-cold 100% methanol (Sigma-Aldrich) for 15 min at −20 °C. After blocking in 1% BSA for 1 h at room temperature, cells were incubated with primary antibody overnight at 4 °C. Dilutions of primary antibodies were in accordance with the manufacturer’s instructions. Cells were then incubated in the dark at room temperature for 1 h with corresponding secondary antibodies conjugated to Alexa Fluor 555 (Thermo Fisher Scientific). Lastly, nuclei were stained with Hoechst 33342 reagent (Thermo Fisher Scientific). Slides were then examined under a Zeiss Axio-Imager Z1 fluorescent microscope (Carl Zeiss International, Germany). At least 5 different fields were analyzed at 20× or 63× magnification.

### 2.5. Antibodies 

The following antibodies were used in Western blotting and immunofluorescence: 

phospho-GSK3α/β (CST 8566), phospho-β-Catenin (Ser33/37/Thr41) (CST9561), non-phospho (Active) β-Catenin (Ser33/37/Thr41) (CST 8814), total β-Catenin (CST 9587), phospho-cyclin D1 (CST 3300), PPARγ (CST 2443), p27 (CST 2552), Ki-67 (CST 9129), phospho-c-Jun (CST 9164), c-Myc (CST 13987), Cytochrome c (CST 4280),GFAP (CST 3670), vimentin (CST 5741), NF-kB (CST 8242), c-Fos (CST 2250) from Cell Signaling Technology (Danvers, MA, US); Lamin B1 (10H34L18), Cyclin D1 (MA5-14512), Bcl-2 (13-8800), phospho-CREB (MA1-083), Ki-67 (MA5-14520) from Invitrogen, Thermo Fisher Scientific, and Phospho-JNK (sc-6254) from Santa Cruz Biotechnology (Dallas, TX, USA).

### 2.6. Cell Viability, Cytotoxicity, Apoptosis Assay

Cell viability, cytotoxicity, and apoptosis were measured in transduced cells by using the ApoTox-Glo Triplex Assay (Promega, Madison, WI, USA) according to the manufacturer’s instructions. Briefly, 48 h after transduction, cells were puromycin-selected for 48 h and 8.0 × 10^4^ cells were seeded per well in a 96-well plate. Following 1-h incubation with viability/cytotoxicity reagent, fluorescence was measured by using the GloMax Discover Microplate Reader (Promega) set on 400Ex/505Em for viability and on 485Ex/520Em for cytotoxicity. Then, Caspase-Glo 3/7 Reagent was added and incubated for 30 min. Luminescence was measured to assess apoptosis in the samples.

### 2.7. Statistical Analysis

Statistical analysis was carried out using Graph Pad Prism 5.0 software (San Diego, CA, USA) by applying the two-way ANOVA and Sidak post-test. The differences were considered significant with *p* < 0.05 * and *p* < 0.01 **.

## 3. Results

### 3.1. Lamin B1 Accumulation Determines GSK3β Inactivation, but Not the Upregulation of β-Catenin Targets

**Figure 2:**
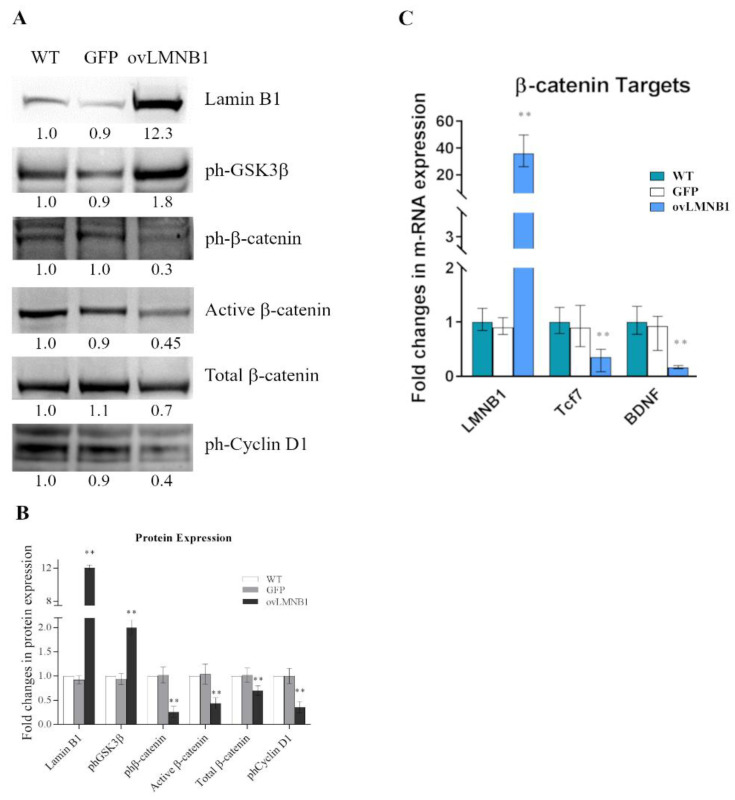
Lamin B1 overexpression affects the activity of GSK3β and the phosphorylation of its targets: (**A**): The expression and the phosphorylation of targets of GSK3β were evaluated in U87-MG cells 96 h after transduction. Lamin B1-overexpressing cells (ovLMNB1) were compared to wild type (WT) and mock-transduced (GFP) samples. The expression or phosphorylation of the denoted proteins was assayed. Samples were normalized by Total Protein Normalization and compared to WT sample. Western blot results are representative of five independent experiments. (**B**): Quantitative analysis of protein expression, wild type cells (WT) were used as reference sample. ** denotes *p* < 0.01 vs. corresponding mock-transduced sample (GFP). (**C**): The expression of β-catenin targets was tested in cells transduced to overexpress Lamin B1 (ovLMNB1) and compared to wild type (WT) and mock-transduced (GFP) cells. Cells were tested after 48 h of puromycin selection, i.e., 96 h after transduction overall. The panel shows the evaluation of mRNA levels by real-time PCR. GAPDH was used as a housekeeping gene and wild type cells as a reference sample. All the analyses are from five independent experiments, with ** *p* < 0.01 vs. corresponding mock-transduced sample (GFP).

In the astrocytic cell line U87-MG, Lamin B1 overexpression determines an increase in glycogen synthase kinase 3β (GSK3β) phosphorylation, hence it is associated with GSK3β inactivation [17]. In order to investigate the effects of reduced GSK3β activity, we examined the phosphorylation levels of its target β-catenin (Figure 2A). The increase in phosphorylated GSK3β in the sample overexpressing Lamin B1 is matched by a reduction in the amount of β-catenin phosphorylated on Ser33, Ser37, and Thr41 that represent GSK3β’s phosphorylation sites. However, the decrease in phosphorylated β-catenin does not correspond to an increase in its active form, since the amount of non-phosphorylated β-catenin is lower in cells overexpressing Lamin B1 compared to wild type and mock-transduced (GFP) cells. The lack of an increase in β-catenin activation corresponds to an overall decrease in total β-catenin levels and thus to an overall inactivation of the β-catenin pathway observed in Lamin B1-overexpressing cells. Moreover, in the cells that overexpress Lamin B1, GSK3β inactivation is associated with a decrease in the amount of cyclin D1 phosphorylated on Thr286.

Next, we evaluated the mRNA levels of some well-known β-catenin targets (Figure 2C) in order to determine whether the reduction in active β-catenin affects the expression of its targets. The cells that overexpress Lamin B1 show a statistically significant decrease in mRNA levels of the transcription factor Tcf7 and also of the neurotrophin BDNF compared to wild type and mock-transduced (GFP) cells, confirming that Lamin B1 accumulation is associated with a reduction in β-catenin pathway activation.

### 3.2. Lamin B1 Build Up Affects Proliferation and Cell Cycle Progression

**Figure 3:**
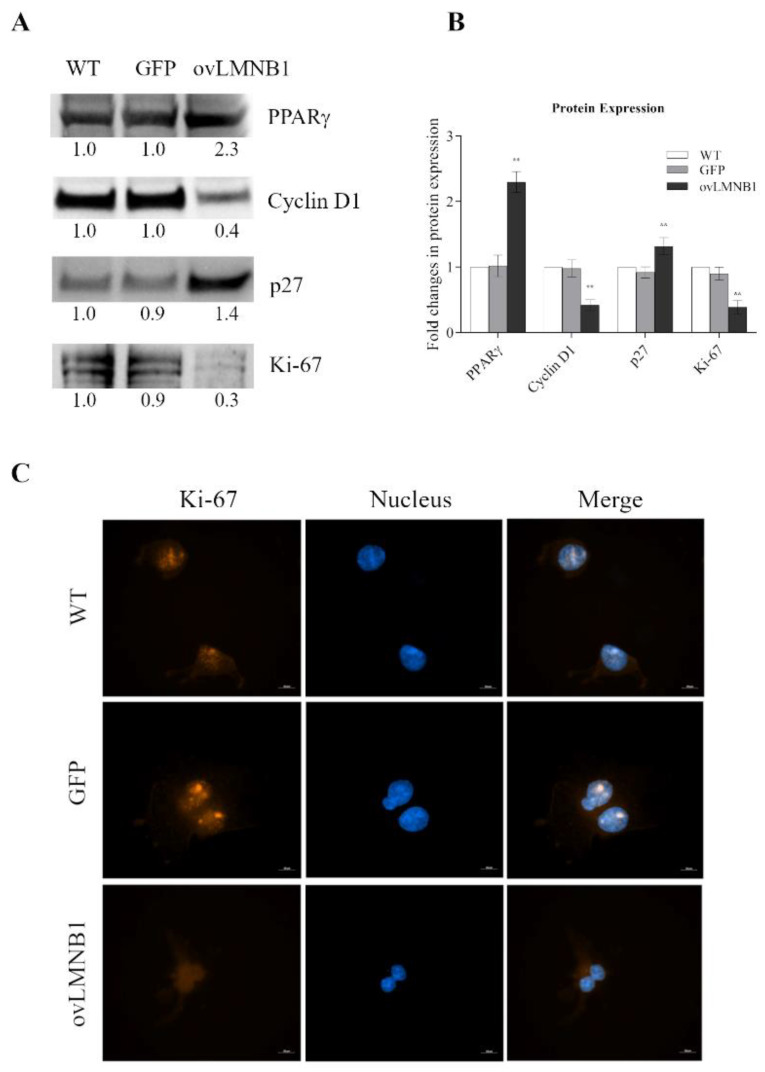
Lamin B1 build up inhibits cell cycle progression and proliferation: The expression of proteins involved in cell cycle progression and apoptosis was evaluated in U87-MG cells 96 h after transduction. Lamin B1 overexpressing cells (ovLMNB1) were compared to wild type (WT) and mock-transduced (GFP) samples. (**A**): Western blot results representative of five independent experiments. The expression of the denoted proteins was assayed. Samples were normalized by performing total protein normalization vs. wild type cells. (**B**): Quantitative analysis of protein expression, wild type cells (WT) were used as reference sample. ** denotes *p* < 0.01 vs. corresponding mock-transduced sample (GFP). (**C**): Immunofluorescence staining of Ki67 at 63× magnification (bar: 10 µM) in wild-type (WT), mock-transduced (GFP), and Lamin B1-overexpressing (ovLMNB1) cells. Nuclei were stained using Hoechst 33342 (blue). The staining of Ki67 is marked in WT and GFP samples, while it becomes faint in cells overexpressing Lamin B1. Results are representative of at least five different fields.

Next, we investigated whether Lamin B1 accumulation affects the expression of the nuclear receptor Peroxisome Proliferator-Activated Receptor γ (PPARγ). Figure 3A displays that cells overexpressing Lamin B1 show a marked increase of PPARγ expression compared to control samples (WT and GFP), confirming the feature of the β-catenin pathway and PPARγ to also work in opposition in our model, proving that Lamin B1 build up induces the expression of PPARγ.

Moreover, PPARγ can affect cell cycle progression both by inducing the expression of cyclin-dependent kinase inhibitor p27 and by decreasing cyclin D1 expression through reducing the cytosolic β-catenin accumulation. Therefore, we evaluated the expression of both p27 and cyclin D1. Figure 3A illustrates that cells overexpressing Lamin B1 show a marked decrease of cyclin D1 paralleled by a pronounced increase of p27, compared both to wild type and mock-transduced (GFP) cells, indicating a block in cell cycle progression following Lamin B1 accumulation.

In order to determine whether Lamin B1 build up is also associated with an inhibition of cell proliferation, we evaluated the expression of the proliferation marker Ki-67 by Western blot (WB) and immunofluorescence (IF). Both WB and IF (Figure 3A,C) revealed that the expression of Ki-67 is markedly reduced in cells with Lamin B1 overexpression compared to wild type and mock-transduced (GFP) cells. Still, wild type and GFP samples show comparable levels of Ki-67. Therefore, cell proliferation is negatively affected by Lamin B1 accumulation.

### 3.3. Lamin B1 Accumulation Reduces Cell Viability and Causes the Induction of Apoptosis

**Figure 4:**
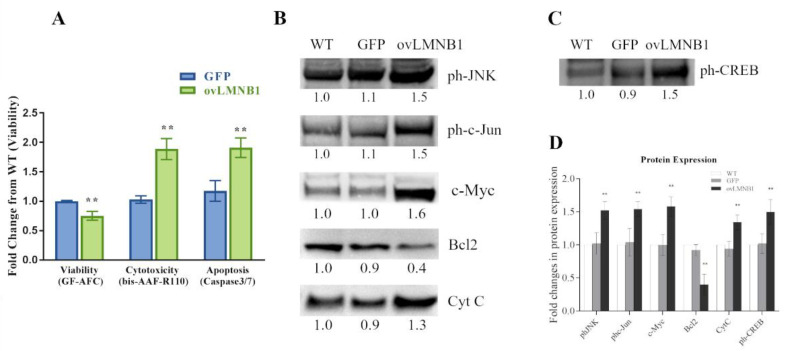
Lamin B1 accumulation reduces cell viability and induces apoptosis signaling pathways: (**A**): Cell viability, cytotoxicity, and apoptosis of transduced cells (ovLMNB1 and GFP) were evaluated by using the ApoTox-Glo Triplex Assay and compared to wild type cells. Analyses from three independent experiments, with ** *p* < 0.01 vs. corresponding mock-transduced sample (GFP). (**B**): The activation of signaling pathways involved in apoptosis and the expression of downstream targets was evaluated in U87-MG cells 96 h after transduction. The expression or the phosphorylation of the denoted proteins were evaluated. (**C**): CREB phosphorylation was measured in wild type, mock-transduced, and Lamin B1--overexpressing cells. Samples were normalized by using total protein normalization vs. wild type cells. Results are representative of five independent experiments. (**D**): Quantitative analysis of protein expression; wild type cells (WT) were used as reference sample. ** denotes *p* < 0.01 vs. corresponding mock-transduced sample (GFP).

Next, we evaluated the effects of Lamin B1 build up on cell viability. Figure 4A demonstrates that cells overexpressing Lamin B1 have a statically significant lower viability compared to mock-transduced cells (GFP). Moreover, cells transduced to overexpress Lamin B1 show higher levels of both cytotoxicity and apoptosis in respect to mock-transduced cells (GFP). These differences are all statistically significant and indicate that Lamin B1 overexpression has a cytotoxic effect and promotes apoptosis. 

In order to further investigate the induction of the apoptotic process, we explored the activation of c-Jun N-terminal kinase (JNK) signaling pathway. Figure 4B shows that cells overexpressing Lamin B1 bear higher levels of phosphorylated, and thus activated, JNK that, in turn, determines higher levels of phosphorylated c-Jun compared to control samples (WT and GFP). Likewise, the expression of the proto-oncogene c-Myc is induced in Lamin B1-overexpressing cells in respect to wild type and mock-transduced (GFP) cells, which, in a context of cell cycle arrest, is also associated with the induction of apoptosis. Finally, Bcl2 expression is lower in cells overexpressing Lamin B1 compared to controls and, as a result, cytochrome C is increased in response to Lamin B1 accumulation. Therefore, Lamin B1 accumulation induces the activation of the JNK-signaling pathway and determines the induction of apoptosis.

Nevertheless, Figure 4C illustrates that cells overexpressing Lamin B1 show a tentative activation of survival pathways, evidenced by the increase in CREB phosphorylation compared to control samples, which does not result in an increase in the expression of downstream effectors of the survival pathway, as proved by the decrease in Bcl2 expression in Lamin B1-overexpressing cells compared to controls (WT and GFP). Therefore, the increase in CREB phosphorylation observed following Lamin B1 overexpression does not correspond to an induction of pro-survival downstream mediators.

### 3.4. Lamin B1 Overexpression Induces Reactive Phenotype in Astrocyte-like Cells

Since Lamin B1 build up determines alterations in cell proliferation and cell cycle progression, and results in increased cell death, we investigated the induction of markers of reactive astrocytes. Wild type, mock-transduced (GFP), and Lamin B1 overexpressing cells were tested in immunofluorescence (IF) and in Western blot for glial fibrillary acidic protein (GFAP) and vimentin expression.

**Figure 5:**
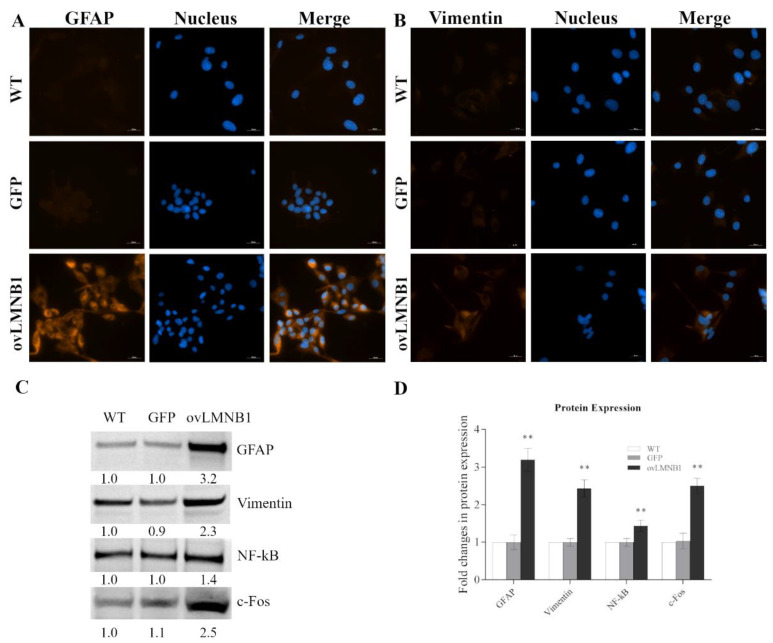
Lamin B1 accumulation induces the expression of markers of reactive astrocytes: The expression levels of reactive astrocytes markers were evaluated in U87-MG astrocyte-like cells transduced with Lamin B1-coding vector (ovLMNB1) and compared to wild type (WT) and mock-transduced cells (GFP). (**A**,**B**) display immunofluorescence staining of GFAP and vimentin, respectively, at 40× magnification (bar: 20 µM). Nuclei were stained using Hoechst 33342 (blue). Results are representative of at least five different fields. (**A**,**B**): In WT and GFP samples, only background fluorescence is seen for GFAP and vimentin, while the signal is strong in ovLMNB1 samples. (**C**): GFAP, vimentin, nuclear NF-kB, and c-Fos expression was measured in wild type, mock-transduced (GFP), and Lamin B1-overexpressing cells. Samples were normalized by using total protein normalization vs. wild type cells. Results are representative of three independent experiments. (**D**): Quantitative analysis of protein expression; wild type cells (WT) were used as reference sample. ** denotes *p* < 0.01 vs. corresponding mock- transduced sample (GFP).

IF revealed an increased immunoreactivity for both GFAP and vimentin (Figure 5A,B) in cells overexpressing Lamin B1 compared to controls (WT and GFP), where the staining for these two proteins is almost undetectable.

Moreover, whole cell lysates were tested for GFAP and vimentin expression while nuclear extracts were probed for phosphorylated NF-kB to assay its nuclear expression in Lamin B1-overepressing cells (ovLMNB1) and in control samples (WT and GFP) (Figure 5C). Western blot analysis shows an increase in GFAP and vimentin expression confirming IF data as well as a rise in phosphorylated NF-kB nuclear localization. The increase in GFAP and vimentin expression associated with the increase in NF-kB phosphorylation are all markers of reactive astrocytes, therefore Lamin B1 build up determines the induction of reactivity.

Likewise, the immediate early response gene c-Fos shows a marked increase in the sample that overexpresses Lamin B1 compared to the controls, demonstrating that Lamin B1 overexpression is also associated with a substantial cellular activation (Figure 5C).

## 4. Discussion

**Figure 6:**
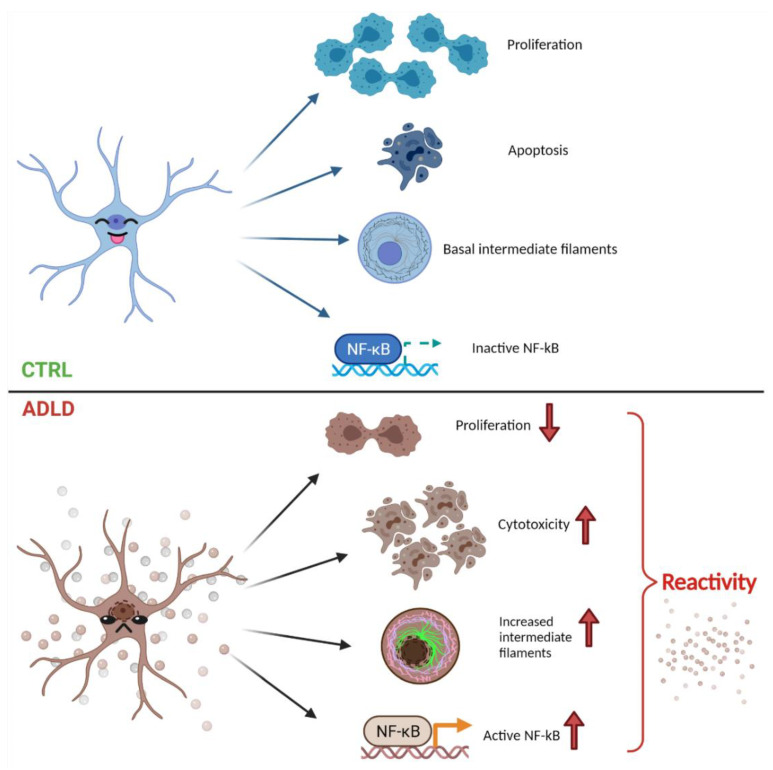
Lamin B1 accumulation causes astrocytes’ reactivity: Lamin B1 build up affects proliferation and cell cycle progression. These events are also associated to a reduction of cell viability and an induction of apoptosis, suggesting a great sufferance of this specific cell population under the effect of Lamin B1 accumulation. Moreover, astrocyte-like cells overexpressing Lamin B1 show increased immunoreactivity for both GFAP and vimentin, together with NF-kB phosphorylation, suggesting the induction of astrocytes reactivity and cellular activation. Image created with Biorender.com.

ADLD is a rare adult-onset neurodegenerative disorder characterized by LMNB1 alterations and with no effective therapies. The clinical phenotype mainly links the pathological phenotype to the overexpression of Lamin B1 protein due to LMNB1 duplication or to LMNB1 upstream deletion [32]. Indeed, LMNB1 alterations have been related also to other neurological diseases such as Parkinson’s, Alzheimer’s, and Huntington’s diseases [33,34,35]. These evidences strengthen the relevant role of Lamin B1, not only in the structural support of the nuclear envelope, but also in relation to many cellular mechanisms [36]. Interestingly, LMNB1-associated disorders seem to share the fact that the Lamin B1 level of expression (either increased or decreased in different disease models) is responsible for the pathological phenotype, suggesting a very close relation between a morphological, physical, and functional balance [35]. Several new evidences also suggest that Lamin B1 levels can be relevant for cell migration mechanisms, neurogenesis control, and cellular aging [37,38,39]. Stemming from these reflections, our astrocytic in vitro ADLD cellular model highlighted new molecular and functional insights that could give new clues to this biological and pathological intriguing puzzle. Indeed, in our previous work, we demonstrated the pivotal role of astrocytes in ADLD pathogenesis [15], and these new data suggest that Lamin B1 accumulation determines different relevant physio-pathological alterations in cell survival, cycle regulation, proliferation, viability, toxicity, and apoptosis. This in vitro experimental model clearly presents limitations related to the tumoral characteristic of the cell line, but it represents a good and reliable model to analyze experimental properties otherwise difficult to evaluate, considering the extremely rare nature of the disease. In our previous work [15], we demonstrated that Lamin B1 overexpression in the astrocytic cell line U87-MG determines an increase in glycogen synthase kinase 3β (GSK3β) phosphorylation, hence it is associated with GSK3β inactivation. In order to investigate the effects of reduced GSK3β activity, we examined the phosphorylation levels of its most studied target, i.e., β-catenin. Interestingly, Lamin B1 accumulation determines GSK3β inactivation, but not the upregulation of β-catenin targets, such as the transcription factor Tcf7 and the neurotrophin BDNF. Therefore, Wnt/β-catenin brain survival signaling pathways appear to be downregulated after Lamin B1 accumulation. Moreover, GSK3β inactivation is associated with a decrease in the amount of cyclin D1 phosphorylated on Thr286, which can affect its nuclear export and thus cell cycle progression. Considering also the well-established crosstalk between β-catenin pathway and the nuclear receptor Peroxisome Proliferator-Activated Receptor γ (PPARγ) [20] and the pivotal role of PPARγ in the central nervous system [40], we investigated whether Lamin B1 accumulation affects the expression of this protein. Our ADLD model showed a noticeable increase in PPARγ expression compared to control samples linked to a pronounced increase in p27 and a clear decrease in cyclin D1. These data, together with the reduction of the proliferation marker Ki-67, indicate that Lamin B1 build up is associated to a block in cell cycle progression. In addition, our engineered ADLD in vitro model showed a decrease of cell viability and an increase in cytotoxicity and apoptosis due to Lamin B1 overexpression, characterized by the activation of the JNK-signaling pathway. Surprisingly, the ADLD model overexpressing Lamin B1 showed a tentative activation of survival pathways denoted by the increase in cAMP-response element binding protein (CREB) phosphorylation compared to control models. However, it does not result in the activation of downstream effectors of the survival pathway, as proved by the decrease in Bcl2 and by the evidence of cellular apoptosis in the ADLD model. The critical role of CREB in different cancers and also in CNS disorders has indeed highlighted how many complex and controversial mechanisms underline the pathological events of different diseases [26,41]. All these new data point out the effect of Lamin B1 accumulation in several pivotal cellular mechanisms that could be underneath the pathological progression of ADLD disease in relation to the astrocyte function. Considering the observed mechanisms that underline an overall adverse effect of Lamin B1 accumulation on the astrocytes, we decided to investigate astrocytes’ reactivity in response to Lamin B1 overexpression. Indeed, reactive astrocytes have been related to many debated pathological mechanisms in the CNS in relation to several injuries or diseases [42]. More specifically, many neurodegenerative disorders such as Alzheimer’s, Huntington’s, Parkinson’s, ALS, and Multiple Sclerosis diseases have been associated with reactive astrocytes’ activity [42]. The scientific community is still debating the correct nomenclature, definitions, and future directions of reactive astrocytes underlying the complexity underneath these topics, as reactive astrocytes undergo several morphological, molecular, and functional changes induced by CNS injuries, diseases, and infections [30]. What is certain is that reactive astrocytes could influence disease outcomes acting on many different physio-pathological mechanisms and that are characterized by morphological changes and molecular changes, with the overexpression of intermediate filaments proteins such as GFAP or Vimentin, and a reduction of cell migration and proliferation [31]. Our ADLD astrocyte-like model overexpressing Lamin B1 shows increased immunoreactivity for both GFAP and vimentin, together with alterations in cell proliferation and cell cycle progression. NF-kB is phosphorylated after Lamin B1 accumulation, indicating NF-kB activation in astrocytes overexpressing Lamin B1. The increase in GFAP and vimentin expression associated with the increase in NF-kB activation are all markers of reactive astrocytes [31], suggesting, for the first time, that Lamin B1 accumulation could trigger astrocytes reactivity (Figure 6). In addition, our ADLD model showed that the marked increase of an early response gene, c-Fos, demonstrates that Lamin B1 overexpression is associated also with a substantial cellular activation [43]. Considering also the possible role of PPARs in the metabolic and inflammatory adaptation of reactive astrocytes [44], it is possible that Lamin B1 accumulation in astrocytes could induce a reactive phenotype associated with the described alterations in cell survival, cycle regulation, proliferation, viability, toxicity, and apoptosis. All in all, these data suggest that Lamin B1 accumulation is associated with astrocyte-like cells biochemical, metabolic, and morphologic remodeling, probably related to the induction of reactive astrocytes phenotype that could be directly associated to ADLD pathological mechanisms. Together with our previous published data [15], these new evidences support the hypothesis that astrocytic dysfunction could not only reduce the support to the other cellular populations involved in the myelination process, but also induce several pathological direct alterations able to trigger the disease phenotype. Therefore, the understanding of specific CNS cell populations’ alterations could help to understand the still unknown morphological and molecular mechanisms involved in the development of ADLD.
